# SerpinB3/Protease Activated Receptor‑2 Axis Is Essential for SARS CoV‑2 Infection

**DOI:** 10.1021/acsinfecdis.5c00145

**Published:** 2025-05-23

**Authors:** Ilaria Frasson, Santina Quarta, Mariagrazia Ruvoletto, Alessandra Biasiolo, Monica Chinellato, Cristian Turato, Maristella Maggi, Laura Cendron, Sara N. Richter, Patrizia Pontisso

**Affiliations:** † Department of Molecular Medicine, University of Padua, via Gabelli 63, 35128 Padua, Italy; ‡ Department of Medicine, University of Padua, via Giustiniani, 2, 35128 Padua, Italy; § Department of Molecular Medicine, University of Pavia, Viale Golgi 19, 27100 Pavia, Italy; ∥ Department of Biology, University of Padua, via Bassi 58/B, 35121 Padua, Italy; ⊥ Microbiology and Virology Unit, Padua University Hospital, Padua 35128, Italy

**Keywords:** SerpinB3, Protease Activated Receptor 2, SARS-CoV-2 infection, Spike, HepG2 cells, Calu-3

## Abstract

Recent research has proposed several host factors required for SARS-CoV-2 infection and involved in the inflammatory response. Among these, members of the human serpin family and PAR2 have been suggested to play a relevant role. As it has been shown that one of the multiple activities of protease inhibitor SerpinB3 is the activation of PAR2, we have modulated the expression of these two molecules on both human bronchial and hepatic cells and assessed cell surface Spike binding and SARS-CoV-2 infectivity. Our findings indicate that both SerpinB3 and PAR2 play a pivotal role in viral infection and downregulate the expression of interferon-γ, a cytokine with a well-known antiviral effect. These results underscore the potential of the SerpinB3-PAR2 axis as a target for antiviral therapy and provide support for addressing serpins as targets for this purpose.

## Graphical abstract

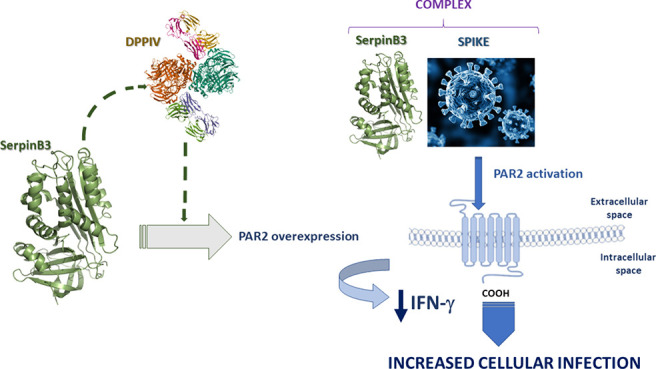

## Introduction

Serpins (serine protease inhibitors or classified inhibitor family I4) are a large family of protease inhibitors involved in the control of several physiological processes, including coagulation, fibrinolysis, inflammation, and angiogenesis.^1^ The members of the Serpins superfamily are broadly distributed, with more than 1500 members identified in humans, viruses, bacteria, and plants.^2^ Thus, far, 37 Serpins have been identified in human cells, with 30 members characterized by functional antiprotease activity.^3,4^ Serpins vary in molecular weight (from 40 kDa up to 100 kDa), but their core structure is extremely conserved, as it is crucial for their function.^4^ The key conserved feature is the flexible reactive center loop (RCL), positioned on top of the folded protein, that contains the enzyme cleavage site. The active site loop of the serpin acts as a bait for cellular proteases. When a protease attempts to cleave this loop, it actually catalyzes the cleavage of the bond between the P1 and P1′ residues within the serpin itself, triggering the inhibition mechanism. The cleavage process can either release serpin from the inactive protease–serpin complex or the two proteins remain bound in a covalent complex.^1,4^

SerpinB3 (SB3) is a member of the vast human serine–protease inhibitor family, physiologically expressed at the basal level in normal epithelial cells and overexpressed in cancer cells and airway tissue.^5^ In the liver, SB3 and its related isoform SerpinB4 (SB4) are undetectable in normal hepatocytes, but their expression increases in response to oxidative stress damage and disease progression.^6^ SB3 is a pleiotropic molecule that, besides its antiprotease activity, has been profoundly involved in liver fibrosis^7^ and inflammation.^8^ These features were also confirmed in a mouse model of lung fibrosis, where SB3 overexpression induced an abnormal expression of inflammatory molecules and more severe lung fibrosis.^9^ In addition, SB3 has been found in the lung tissue of patients with cystic fibrosis.^10^ Notably, SB3 has also been implicated as a positive cofactor in the progression of HCV and HPV infection and related malignancies.^11,12^

SARS-CoV-2, the causative agent of COVID-19, is a positive-sense RNA betacoronavirus belonging to the Coronaviridae family, along with other respiratory coronaviruses that infect humans.^13^ The typical critical illness related to SARS-CoV-2 infection is characterized by hypoxemic respiratory failure and acute lung injury. Almost five years after the onset of the COVID-19 pandemic, numerous viral variants have emerged, and many aspects of the individual response to the SARS-CoV-2 infection remain unclear. The major SARS-CoV-2 variants include the original Wuhan variant and its associated D614G mutation, the Delta variant known for its significantly higher transmissibility and severe disease, and the Omicron variant, which is characterized by increased transmissibility and potential immune escape. These variants emerged independently, and each rapidly became dominant, either regionally or globally, overtaking previous variants. Recent research has identified host factors that support viral infection^14–16^ and the differences in the host–virus interactions between the major variants. First, the angiotensin-converting enzyme 2 (ACE2) was shown to be the crucial receptor for SARS-CoV-2 infection, determining viral tropism in human tissues.^14^ More recent findings demonstrated that the protease activated receptor 2 (PAR2) is implicated in endothelial dysfunction and orchestrates signaling pathways involved in thrombo-inflammation and apoptosis in patients with COVID-19.^17^ PAR2 is a member of the G-protein coupled surface receptor family profoundly involved in inflammatory response and acts as a sensor of coagulation proteases.^18^ It is worth noting that the antiprotease activity of SB3 was recently found essential for the synthesis and activation of PAR2.^19^

Here we have investigated the role of SB3 alone and in relation to PAR2 in SARS-CoV-2 infection, studying two key viral variants, Wuhan and Omicron, in human lung cells and in hepatoma cell lines, which show different expression levels of SB3. This research aims to understand the molecular basis of the SB3/PAR2 axis involvement in viral infection and the hyperinflammatory immune response that can occur in infected patients.^20^

## Results

### Enhanced Levels of SB3 Boost SARS-CoV-2 Infection in Different Human Cells

As SARS-CoV-2 is known to exploit cell proteases during cell entry, we investigated whether SB3 expression modulated this process. HepG2 cells were reported to express negligible basal levels of SB3 ^21^ and, although they express both the ACE-2 receptor and the cellular transmembrane serine protease TMPRSS2,^22,23^ this cell line has been reported to be poorly susceptible to SARS-CoV-2 pseudovirus infection.^23^ We thus explored the ability of SARS-CoV-2 to infect HepG2 cells using HepG2 cell clones with different levels of SB3 expression. In previous studies, we have established and characterized stably transfected HepG2 cell lines that express high levels of the wild-type human SB3 (HepG2-SB3),^24^ or that express a deleted form of SB3 (HepG2/Δ-SB3) which lacks the antiprotease activity and has a deletion of seven amino acids in the reactive site loop.^25^ This latter isoform serves as a control to study the specific role of SB3′s antiprotease activity, allowing us to distinguish between effects dependent on SB3′s antiprotease function and those arising from properties of the protein itself. SB3-expressing HepG2 cells and HepG2 cells stably transfected with the empty vector (HepG2-CTR), used as the control, were infected with the Wuhan variant of the SARS-CoV-2 virus, and the generation of newly infective viral particles was measured by a virus yield reduction assay (VRA). Upon infection, HepG2-SB3 cells demonstrated a markedly elevated viral load, exhibiting a 2000-fold increase in comparison to the control cells (Figure 1A). In contrast, the viral load in infected HepG2-Δ-SB3 cells was comparable to that of the control cells (Figure 1A).

**Figure 1:**
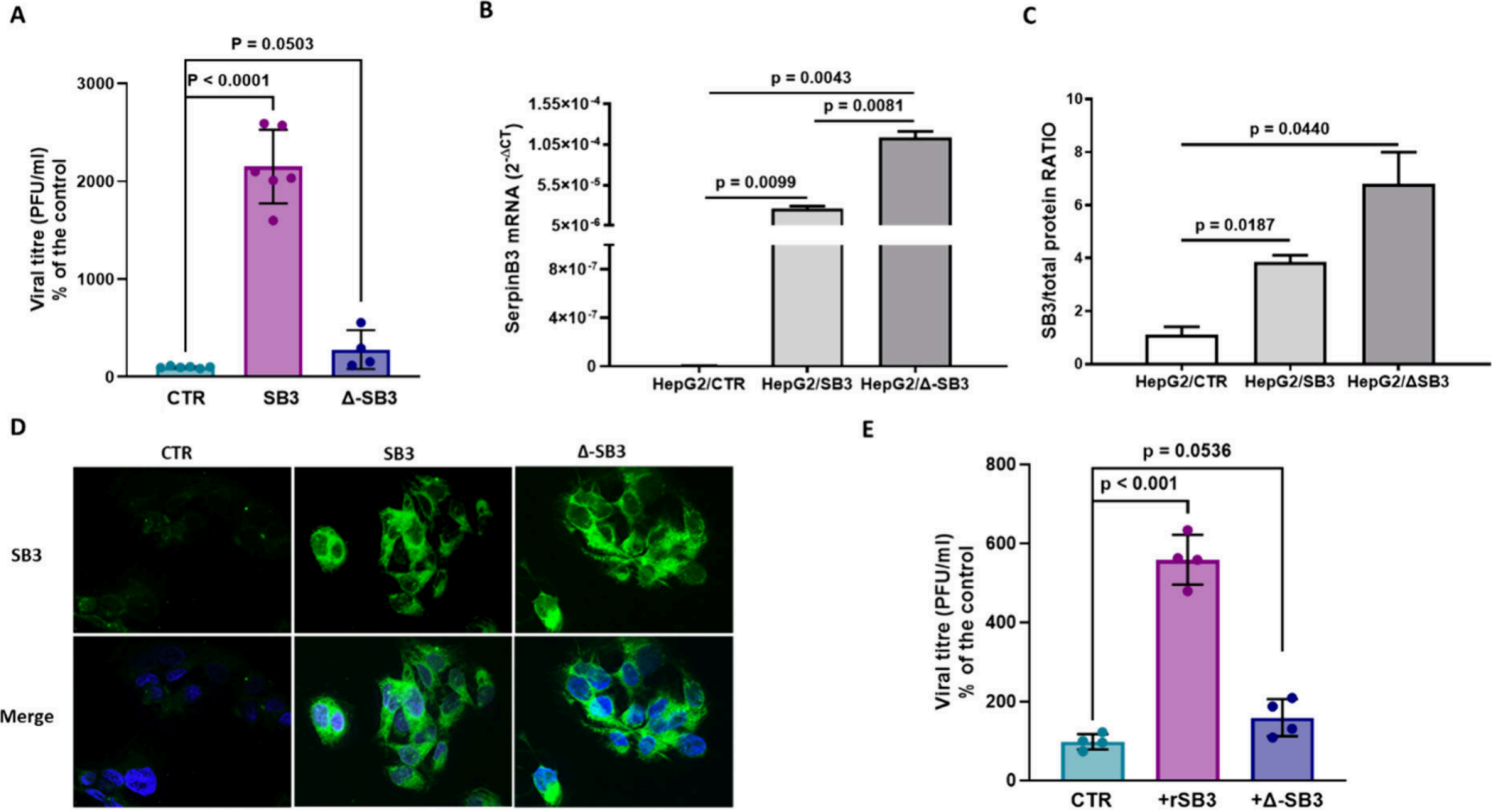
SB3 facilitates SARS-CoV-2 infection in hepatoma cells. A) Efficiency of SARS-CoV-2 infection in HepG2 cells stably transfected to overexpress SB3 (HepG2/SB3) and in cells with a deletion in the reactive site loop of the protein (HepG2/Δ-SB3), with respect to control HepG2 cells, stably transfected with the empty vector alone (HepG2/CTR). The cells were infected with the SARS-CoV-2 Wuhan variant at a multiplicity of infection (MOI) of 0.05, the supernatants were collected at 48 h post-infection and subjected to virus yield reduction assay. The results are expressed as a percentage of plaque-forming units (PFU)/ml in HepG2/SB3 and HepG2/Δ-SB3 cells relative to the controls, and are mean values ± SD of biological replicates, with each dot representing a single data point. Statistical significance was assessed using a two-tailed Student’s *t* test, and p-values are reported. B) SB3 mRNA levels in the different HepG2 clones. The results are expressed as the mean+SEM of gene expression, reported as 2^–Δct^ relative to basal values. C) SB3 protein expression in the different HepG2 clones. The results are expressed as the mean+SEM Ratio of SB3 vs total protein concentration. D) SB3 (green) immunofluorescence results in the different HepG2 clones. Cell nuclei are counterstained with Dapi (blue) and shown as merged images. E) Paracrine effect of recombinant SB3 (rSB3) and recombinant deleted SB3 (Δ-SB3) on SARS-CoV-2 infection in HepG2 cells, stimulated with the recombinant SB3 isoforms for 24 h prior to infection. Data are reported as mean values ± SD of biological replicates, with each dot representing a single data point. Statistical significance was assessed using a two-tailed Student’s *t* test, and all p-values are reported.

To correlate the results of SARS-CoV-2 infection with SB3 and with Δ-SB3 expression, we measured SB3 levels and its cellular distribution in the three different HepG2 cell lines. SB3 expression in cells overexpressing SB3 or ΔSB3 was comparable at both the transcriptional and protein levels (Figure 1B, C, and D). Furthermore, the levels of SB3 expression in HepG2-SB3 and HepG2-Δ-SB3 cells were significantly higher (3-fold and 5-fold, respectively) than in control HepG2 cells. The relative expression levels of SB3 or Δ-SB3 in the different HepG2 cells were measured, using specifically designed primers, and both in HepG2/Ctr and in HepG2/SB3 cells, a similar extent of amplification was achieved using primers encompassing a conserved sequence or the sequence of the deleted site loop, while in HepG2/ΔSB3, amplification was achieved only with primers of the conserved sequence (Figure S1).

In order to evaluate the potential paracrine effect of SB3 in SARS-CoV-2 infection, HepG2 cells, stimulated for 24 h by the administration of the wild type or the deleted form of purified recombinant SB3 proteins (rSB3 or rΔ-SB3), were infected with the SARS-CoV-2 virus. It has previously been shown that the administration of exogenous SB3 enhances SB3 synthesis, via a paracrine mechanism.^24^ Vehicle-treated cells were used as a negative control. Even in this case, cells treated with rSB3, and infected with the SARS-CoV-2 virus, produced higher amounts (6-fold) of new infective particles, compared to control cells (Figure 1E). Notably, cells stimulated with the deleted rΔ-SB3 isoform showed no significant difference in terms of viral load compared with the control (Figure 1E).

As SARS-CoV-2 has been reported to primarily infect the lung tissue, we tested the effect of SB3 expression levels on SARS-CoV-2 infection in Calu-3 cells, which are human bronchial cells susceptible and permissive to SARS-CoV-2.^24^ First, we assessed SB3 basal expression in Calu-3 cells: we observed higher levels of both mRNA and protein expression when compared to HepG2 cells (Figure S2). Subsequently, we monitored SB3 expression and cellular distribution in Calu-3 cells stimulated with exogenous rSB3 for 24 h. The paracrine stimulation by rSB3 did not affect the synthesis and protein expression of endogenous SB3 (Figure 2A,B), while rSB3 determined morphological changes characterized by cellular cluster disaggregation and elongated cellular shape, likely as a result of EMT induction, as previously described in HepG2/SB3 cells.^24^ Next, the SARS-CoV-2 infection was measured in both stimulated and vehicle-treated Calu-3 cells. As expected, Calu-3 cells were highly susceptible to the viral infection; however, rSB3 stimulation further increased their susceptibility to SARS-CoV-2 infection (1.8-fold) (Figure 2C).

**Figure 2:**
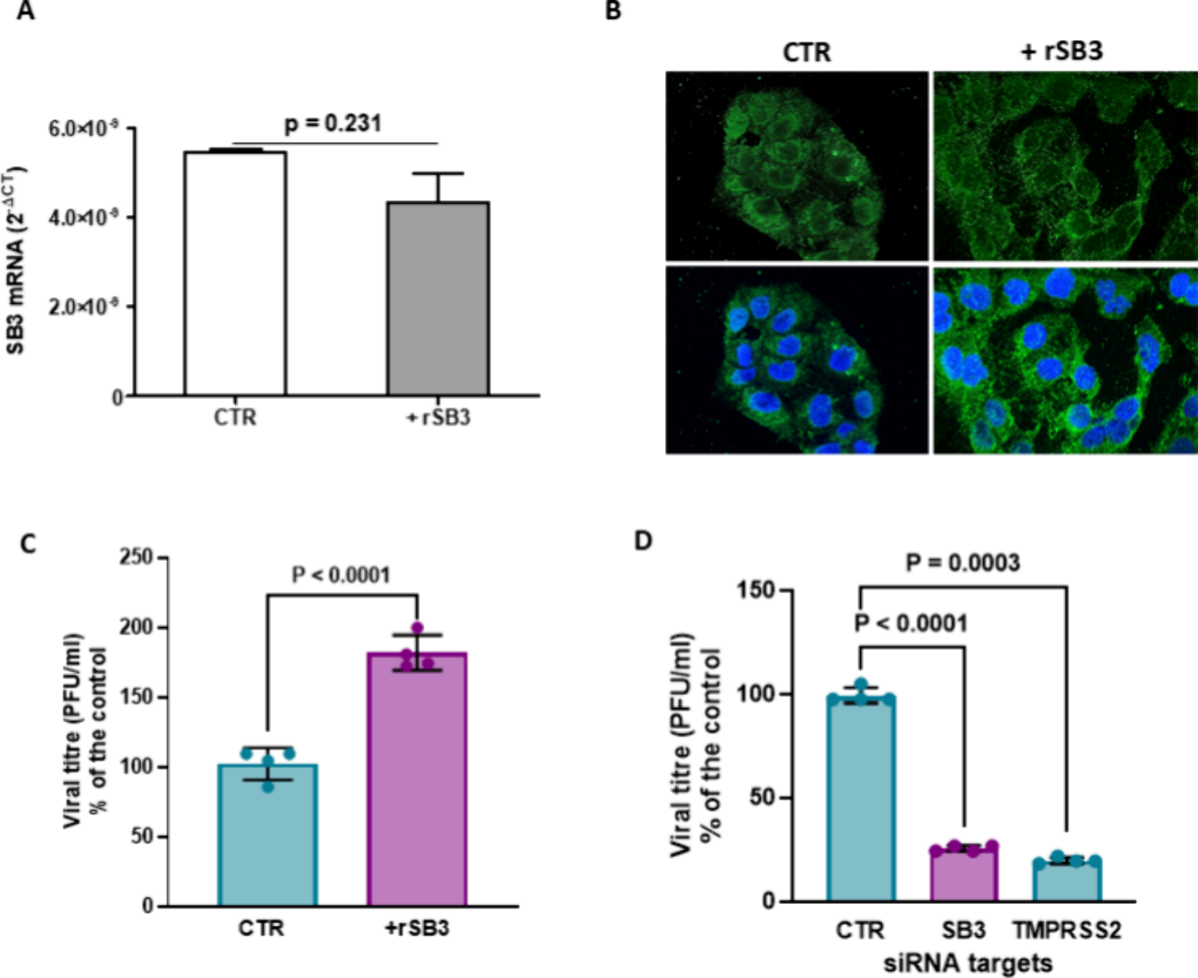
SB3 levels modulate SARS-CoV-2 infection of human bronchial cells. A) SB3 mRNA levels in the human bronchial cells (Calu3 cell line) untreated (CTR) or stimulated with the recombinant SB3 (rSB3) for 24 h. The results are expressed as the mean+SEM of gene expression, reported as 2^–Δct^ relative to basal values. B) Immunofluorescence results of SB3 (green) in Calu-3 cells untreated (CTR) or stimulated with the rSB3 for 24 h. Cell nuclei are counterstained with Dapi (blue) and shown as merge image (lower panels). C) Paracrine effect of rSB3 on SARS-CoV-2 infection in Calu-3 cells. Calu-3 cells were infected with the SARS-CoV-2 Wuhan variant, at a MOI of 0.05 and the production of new infective virus was evaluated 48 h post infection by virus yield reduction assay. Calu-3 cells were untreated (CTR) or stimulated with rSB3 for 24 h prior to infection. D) Effect of siRNA-mediated knockdown of SB3 in Calu-3 cells on viral infection. Calu-3 cells were silenced for SB3 24 h prior to viral infection. TMPRSS2 siRNA-mediated knockdown was used as positive control. Data are reported as mean values ± SD of biological replicates, with each dot representing a single data point. Statistical significance was assessed using a two-tailed Student’s *t* test, and all p-values are reported.

We then further validated the role of SB3 in SARS-CoV-2 infection in human bronchial cells by transient short interfering RNA (siRNA)-mediated knockdown. We downregulated SB3 expression in Calu-3 cells and monitored the efficiency of viral infection. TMPRSS2- knockdown was used as a positive control for inhibition of viral infection, given its role in viral entry.^26^ Mock-transfected cells were used as a negative control, whereas SB3 downregulation was confirmed by RT-PCR (Figure S3).

SB3 knockdown determined an impaired production of new viral particles (approximately 75%), comparable to that achieved with TMPRSS2 knockdown (approximately 80%) (Figure 2D).

Notably, the favorable effect of SB3 on SARS-CoV-2 infection was achieved, despite the possible antiprotease activity of this serpin on TMPRSS2.^26^ To investigate this aspect, we analyzed the SB3 antiprotease activity on TMPRSS2. We observed a dose-dependent inhibition of the TMPRSS2 protease activity by SB3, similar to that obtained by the TMPRSS2 reference inhibitor Camostat.^26^ The dissociation constant for SB3 was 6 times lower than that of Camostat (Figure S4).

These results indicate that the human protein SB3 plays a crucial role as a positive regulator of SARS-CoV-2 infection in human cells, likely involving its antiprotease activity but independently of TMPRSS2 inhibition.

## Interaction between the Viral Spike Protein and SB3

To better understand the mechanism by which SB3 conferred an increased susceptibility to SARS-CoV-2 infection, we used the recombinant Spike protein, since this viral surface protein is involved in viral entry.^26^ The interaction of Spike with the cell surface was assessed by immunofluorescence in unpermeabilized HepG2/CTR cells and in HepG2 cells overexpressing SerpinB3. As shown in Figure 3A, only HepG2/SB3 cells were able to bind Spike, while no fluorescent signal was achieved in HepG2/CTR cells. In addition, overlap of the fluorescence signals of SB3 and Spike was observed (Figure 3B and Figure S5).

**Figure 3:**
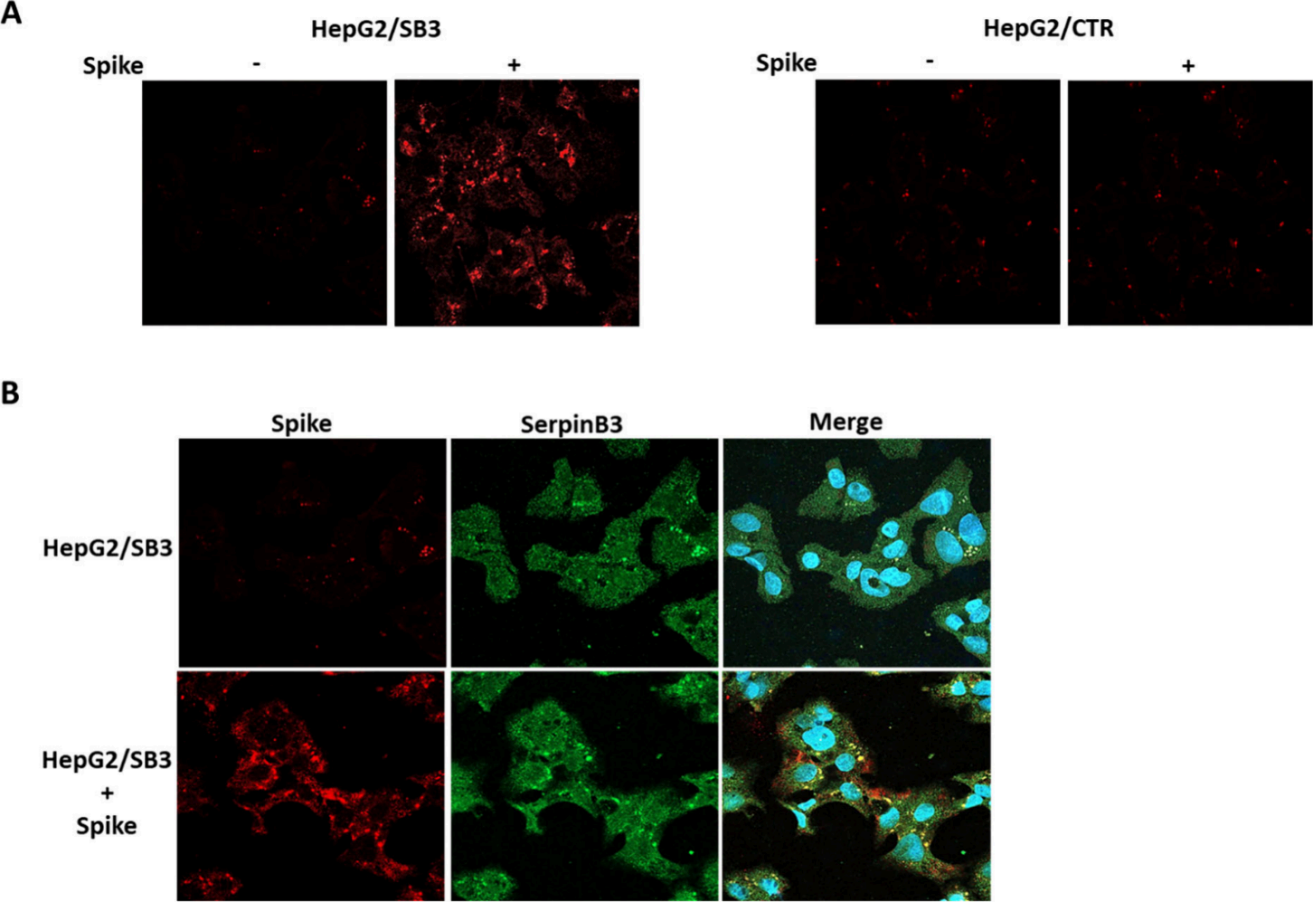
Immunofluorescence analysis for Spike and SB3 in HepG2 cells. A) Surface spike signal (red) in not permeabilized HepG2 cells overexpressing SB3 (HepG2/SB3) cells and in HepG2 control cells (HepG2/CTR) incubated for 2 h with Medium alone (Spike −) or with Spike recombinant protein (Spike + ). B) Confocal immunofluorescence analysis in not permeabilized HepG2/SB3 cells after 2 h treatment with Spike protein. In the left panels Spike (red) and SB3 (green) signals are shown. In the merged image nuclei are counterstained with DAPI (blue). The merged image highlights areas of colocalization (yellow) of Spike and SB3.

These results suggest a potential direct interaction between the two molecules under investigation. However, it cannot be discounted that SB3 induces the expression or modification of another surface molecule with which Spike binds or interacts.

### Interplay between Spike/SerpinB3 Complex and the Cellular Protease Activated Receptor-2

As it has recently been shown that one of the multiple activities of SB3 is the activation of the membrane protease-activated receptor-2 (PAR2),^18^ and this receptor has been proposed to be involved in SARS-CoV-2 infection,^27^ we investigated the possible effect of PAR2 on Spike binding to the surface of cultured cells overexpressing SB3. As shown in Figure 3, when incubated with the recombinant Spike protein, unpermeabilized HepG2 cells overexpressing SB3 showed a remarkable anti-Spike fluorescence signal when compared to controls (Figure 3A,B). In these cells, the fluorescent signal was abolished in the presence of PAR2 siRNA-mediated knockdown (Figure 4A,B). PAR2 fluorescence was abolished not only by PAR2 silencing but also by preincubation of these cells with Spike, which demonstrated a decrease in fluorescence intensity similar to that obtained with PAR2 silencing (Figure 4C,D), suggesting that Spike/SB3 interaction on the cell surface could affect PAR2 antibody recognition.

**Figure 4:**
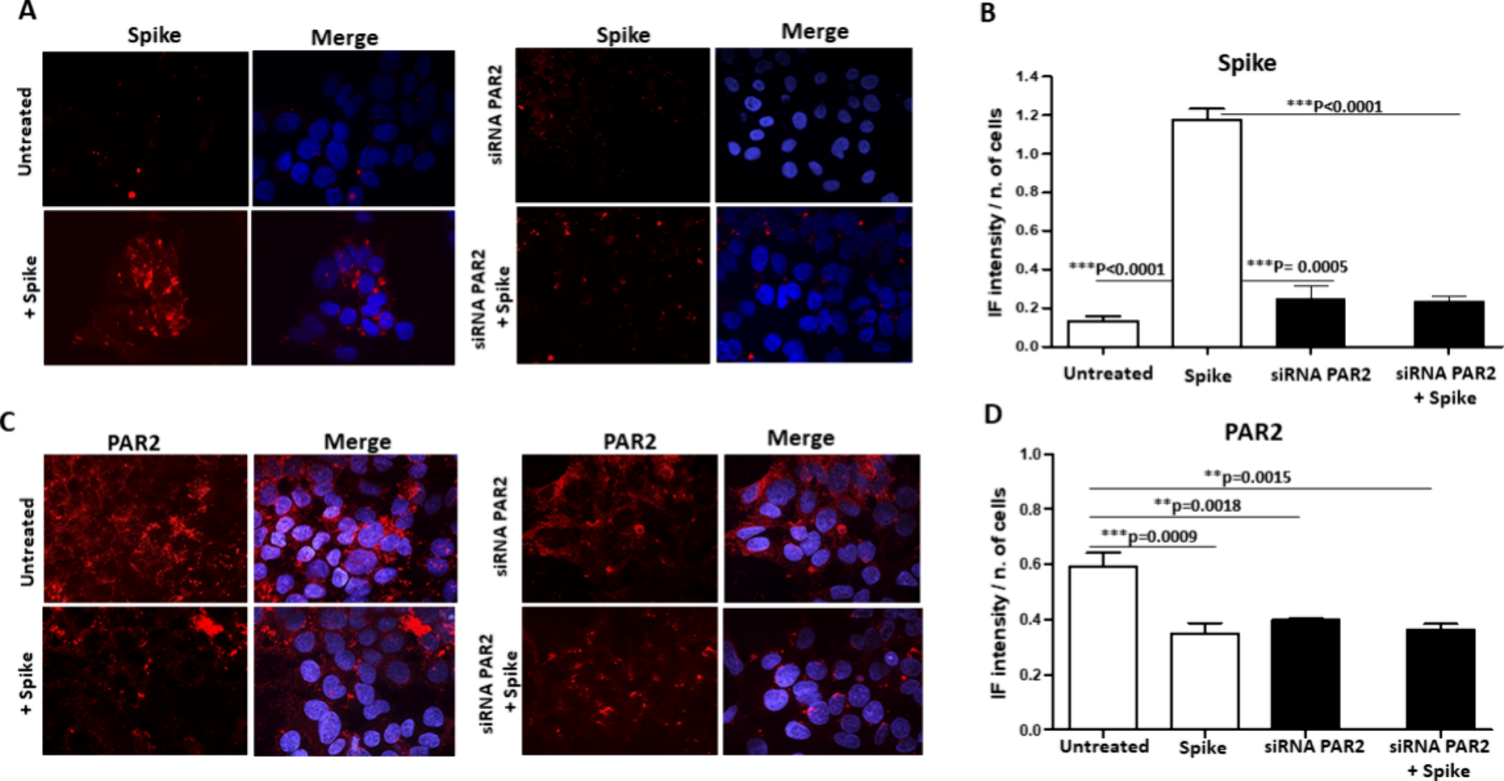
Immunofluorescence analysis for Spike and PAR2 in HepG2 cells overexpressing SerpinB3. A) Immunofluorescence analysis of Spike protein (red) in not permeabilized HepG2/SB3 cells untreated or silenced for PAR2 after 2 h incubation with Spike. In the merged images nuclei are counterstained with DAPI (blue). B) Quantification of Spike protein immunofluorescence signal in HepG2/SB3 cells, expressed as mean fluorescence intensity (MFI) normalized to cell count + SD (Mann–Whitney test). C) Immunofluorescence analysis of PAR2 protein (red) in HepG2/SB3 cells after PAR2 silencing. In the merged images nuclei are counterstained with DAPI (blue). D) Quantification of PAR2 protein immunofluorescence expressed as mean fluorescence intensity (MFI) normalized to cell count + SD (Mann–Whitney test). Only significant p values (p < 0.05) were reported.

It is interesting to note that Spike preincubation and attachment to the cell membrane did not affect PAR2 mRNA levels in both HepG2 cells and Calu-3 cells (Figure 5A), while the activation of the PAR2 signaling cascade, as documented by an increase of phosphorylation of Erk1/2, was slightly higher than that induced by the agonist peptide SLIGKV-NH2 (Figure 5B). Given these results, we investigated whether and how PAR2 expression in human bronchial cells influenced SARS-CoV-2 infection. Calu-3 cells were silenced for the expression of PAR2 (Figure S3) and were infected with both the Wuhan variant and the Omicron variant, i.e., the latest viral variant of concern, of SARS-CoV-2; in both cases, we observed significant reductions in viral infection (approximately 50% and 35%, respectively) (Figure 5C).

**Figure 5:**
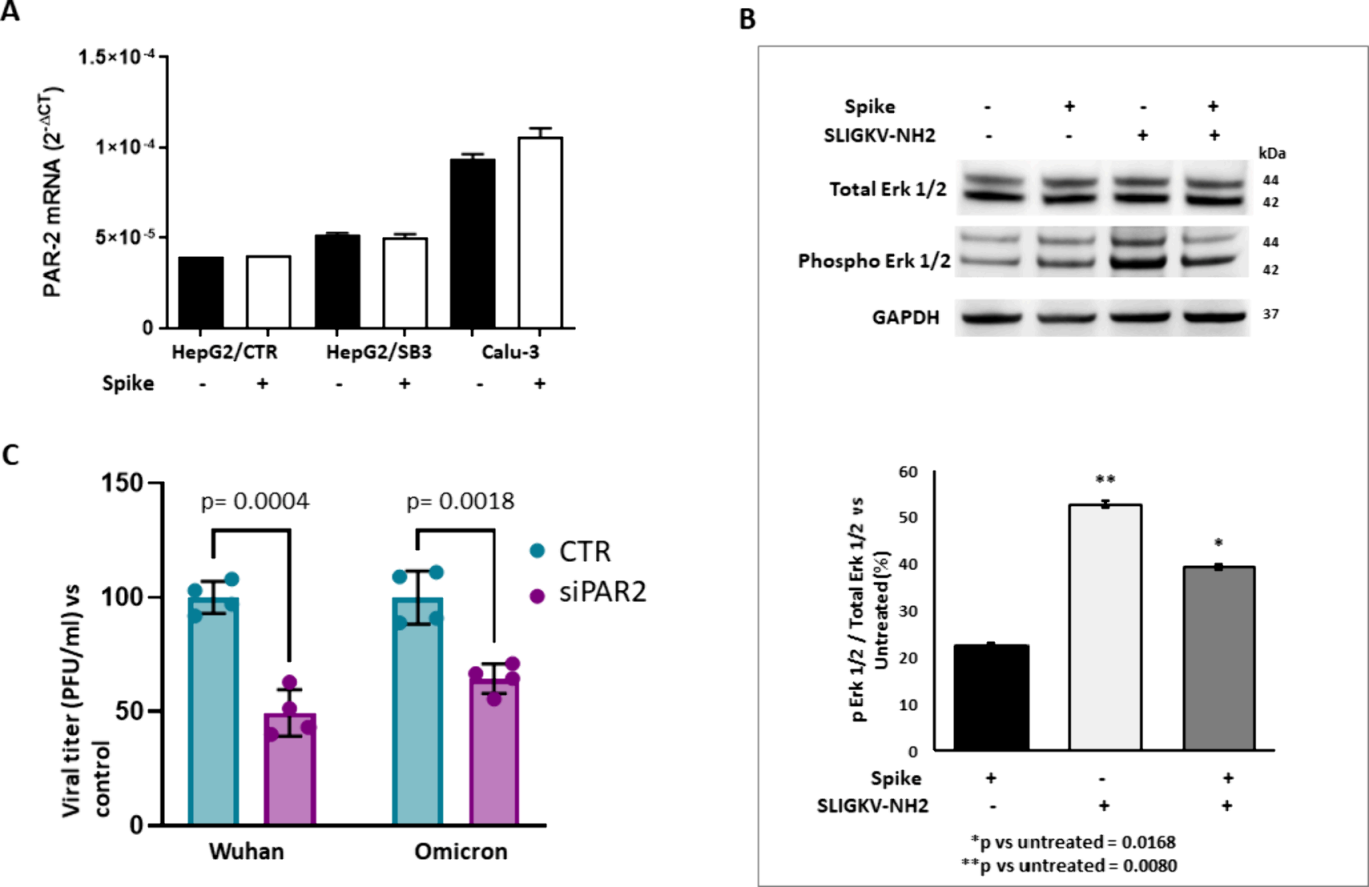
Effect of Spike on PAR2 expression and function and role of PAR2 in SARS-CoV-2 infection. A) PAR2 mRNA expression in different cell lines treated or not with recombinant Spike protein. The results are expressed as the mean+SEM of gene expression, reported as 2^–Δct^ relative to basal values. B) Upper panel: Representative Western Blot analysis of PAR2-induced Erk1/2 phosphorylation in the Calu-3 cell line. Erk1/2 phosphorylation levels were measured after treatment with recombinant Spike protein and the SLIGKV-NH2 activating peptide. Blots display pErk1/2 levels relative to total Erk1/2. GAPDH was used as a loading control. Lower panel: Graph reporting densitometric analysis of Western blots expressed as mean values ± SD of three biological replicates. Data are normalized to GAPDH and expressed as the pErk1/2 to total Erk1/2 ratio vs untreated cells. Statistical significance was assessed by double-tailed *t* test. C) Efficiency of SARS-CoV-2 infection in Calu-3 cells silenced for PAR2 (siPAR2) or not (CTR) and infected with the Wuhan or Omicron variants, at a MOI of 0.05. The generation of novel infectious virus was evaluated at 48 h post-infection by virus yield reduction assay. Graphs report mean values ± SD of at least two biological replicates. Each condition was tested in triplicate per replicate. Individual replicates are shown as dots. The statistical significance was assessed by double-tailed *t* test.

These results demonstrate that PAR2 has a positive regulatory effect on SARS-CoV-2 infection that is potentially mediated by SB3. Importantly, this effect is maintained across different viral variants, suggesting a conserved mechanism of action.

### SB3 and PAR2 Downregulate Interferon-γ Expression

Since interferon-γ (IFN-γ) is a cytokine with a well-known antiviral effect,^28^ we have explored whether both SB3 and PAR2 could affect the expression levels of this cytokine in Calu-3 cells that are susceptible to SARS-CoV-2 infection. As reported in Figure 6A, in control cells IFN-γ was barely detectable, while silencing for both SB3 (Figure 6A) and for PAR2 (Figure 6B) demonstrated a significant increase of this cytokine and a similar effect was also achieved when Calu-3 cells were treated with the PAR2 inhibitor 1-Piperidine Propionic Acid (1-PPA),^29^ where a dose-dependent progressive increase of IFN-γ was observed (Figure 6C).

**Figure 6:**
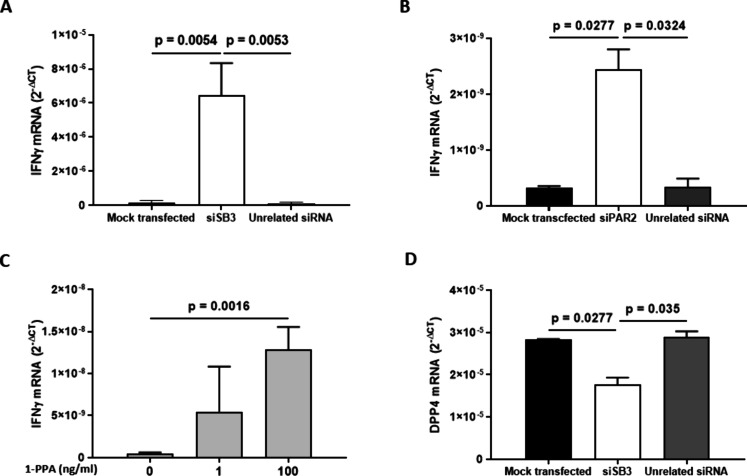
Effect of silencing for SerpinB3 and PAR2 in Calu-3. A) IFNγ mRNA expression in mock-transfected cells and in cells silenced for SB3 (siSB3) or for unrelated siRNA. B) IFNγ mRNA expression in mock-transfected cells and in cells silenced for PAR2 (siPAR2) or for unrelated siRNA. C) IFNγ mRNA expression in Calu-3 cells after 24 h treatment with the PAR2 inhibitor 1-Piperidine-Propionic Acid (1-PPA). The molecular amplification results are expressed as the mean+SEM of gene expression, reported as 2^–Δct^ relative to basal values. The statistical significance was assessed by a Mann–Whitney test.

Since it is known that PAR-2 activation can occur through the soluble dipeptilyl Peptidase-4 (DPP-4)^30^ and this enzyme can be induced by SB3 in HepG2 cells,^31^ we assessed whether silencing SB3 could affect DPP-4 transcription also in Calu-3. Indeed, silencing SB3 abrogated DPP-4 expression (Figure 6D).

Based on these findings, we propose a model where SB3 plays a role in enhancing the SARS-CoV-2 infection of target cells. Our data suggest that SB3 influences the cellular environment, boosting viral infection. This involves interactions with PAR2, leading to its activation. PAR2 activation, in turn, suppresses IFN-γ levels and triggers an inflammatory response, thereby creating conditions that facilitate SARS-CoV-2 infection.

## Discussion

The coronavirus pandemic of 2019 (COVID-19) has resulted in millions of deaths and has caused a global social and economic crisis. Five years after the onset of global SARS-CoV-2 circulation, the critical biological aspects that govern the variability in the human response to viral infection remain to be elucidated. The application of various ’omics’ technologies has led to the rapid discovery of host factors required to sustain the SARS-CoV-2 life cycle.^14,15,32,33^ These studies have highlighted the complex nature of the virus’s entry mechanism, revealing that while ACE2 serves as the primary receptor, SARS-CoV-2 can also exploit accessory receptors and molecules that facilitate viral attachment, internalization, and fusion. However, these findings have not explained which factors determine or are involved in the inflammatory response and relative tissue damage. More recently, the identification of severity-associated activation of the receptor for advanced glycation end products (RAGE) and a strong enrichment in IFN signaling family pathways^34^ has paved the way for new research that considers host factors previously not considered strictly necessary for viral infection.

Members of the human serpin family, ubiquitous proteins with serine protease inhibition functions, have been suggested to play a protective anti-inflammatory role in SARS-CoV-2 infection. The analysis of lung tissue from different donors has identified serpins as proteins that may interfere with viral entry by impeding TMPRSS2-mediated cleavage of the viral spike protein.^35,36^

In this study, we focused on SB3 due to its biological characteristics as a protease inhibitor and as a coplayer in the immune system in malignancies, chronic autoimmune diseases, and HCV infection.^37,38^ With regard to its protease inhibitor function, we have confirmed that SB3 can recognize TMPRSS2 with high affinity, as recently reported.^36^ Nevertheless, its function seems to be more complex than was previously assumed. Our findings indicate that SB3 plays a positive role in SARS-CoV-2 infection in both human bronchial and hepatic cells. The results of this study indicate that increased expression levels of SB3 are associated with a higher susceptibility of human liver and lung cells to SARS-CoV-2 infection. As observed in malignancies,^6^ SB3 overexpression is potentially associated with more severe infection outcomes, due to the observed increased viral replication. These findings are corroborated by a meta-analysis of published transcriptome and proteome profiles of respiratory samples from patients with coronavirus disease 2019 (COVID-19) reporting that SB3 was the most significantly upregulated gene and overexpressed protein in symptomatic patients.^39^

Our research has provided further insight into the role of SB3 in SARS-CoV-2 infection. SB3 is in fact able to induce the activation of protease-activated receptor 2 (PAR2),^19^ a membrane receptor able to regulate host cell interferon and inflammatory cytokines.^28,40^ Indeed, PAR2 has been described as an important host factor in other crucial viral infections, such as influenza A, where it has been shown to inhibit the antiviral response and enhance the inflammatory response.^41–43^

Our results indicate that SB3 facilitates SARS-CoV-2 infection, possibly by direct interaction between SB3 and the Spike protein, even though we cannot exclude that other SB3-induced molecules are involved in the process. The significantly lower viral titer observed in HepG2 cells expressing SB3 lacking its reactive center loop (Δ-SB3) or treated with recombinant Δ-SB3 highlights the importance of this domain in SB3′s effect on viral infection. Our results also point out that SB3 levels influence PAR2 activation, mediating IFN-γ downregulation, thus favoring viral infection.

## Conclusion

Our findings indicate that the expression of SB3 and PAR2 plays a pivotal role in SARS-CoV-2 infection and in the regulation of the inflammatory response to it. Furthermore, they suggest that genetic variations in SB3 expression may serve as a potential determinant of SARS-CoV-2 susceptibility. These results also underscore the potential of the SB3-PAR2 axis as a target for antiviral therapy and provide support for addressing SB3 as a target for this purpose.

## Materials and Methods

### Cell Cultures and Viral Strains

All cell lines were incubated at 37 °C with 5% CO_2_ in a humidified atmosphere. HepG2 (human hepatoma, ATCC HB-8065, Manassas, VA) and VeroE6 (African green monkey, kidney, ATCC CRL-1586) were maintained in Eagle’s Minimum Essential Medium (Merck Sigma-Aldrich, St. Louis, MO, USA). HepG2 cells overexpressing full length SB3 (HepG2/SB3) or SB3 carrying a deletion of 7 aa in the reactive site loop (HepG2/SB3Δ7) and lacking the antiprotease activity^25^ were also used. HepG2 control cells were stably transfected with the empty expression vector (HepG2/Ctr).^24^

The medium used for stably transfected HepG2 clones was supplemented with G418 (Merck Sigma-Aldrich, St. Louis, MO, USA) as a selective agent. Calu-3 cells (Human lung ATCC HTB-55, Manassas, VA) were maintained in DMEM F12 (Merck, Sigma-Aldrich, St. Louis, MO, USA). The human monocytic cell line THP-1 (American Type Culture Collection, ATCC, Manassas, VA) was cultured in Dulbecco’s modified Eagle’s medium (Merck Sigma-Aldrich, St. Louis, MO, USA). All media were supplemented with 10% fetal bovine serum, 100 U/mL of penicillin, 2 mM l-glutamine, and 0.1 mg/mL of streptomycin (Merck Sigma-Aldrich, St. Louis, MO, USA). For seeding and subculturing, cells were washed with phosphate buffered saline (PBS) and then incubated in the presence of trypsin/EDTA solution (Merck, St. Louis, MO, USA) until cells detached.

### Viral Stock Production

To propagate the SARS-CoV-2 isolates, VeroE6 cells were seeded (3.5 × 10^6^) in complete medium (DMEM supplemented with 10% FBS) in T175 vented-cap flasks the day before infection. Complete medium was removed, and cells were infected with the SARS-CoV-2 virus (MOI 0.01) for 1 h at 37 °C in a humidified incubator in serum free fresh medium. The infection medium was removed and replaced with fresh medium (DMEM supplemented with 2% FBS). Supernatants were collected, centrifuged at 2300 rpm for 10 min, and stored in aliquots at −80 °C.

### SARS-CoV-2 Titration by Virus Yield Reduction Assay

Viral particles were titered by the virus yield reduction assay (PRA). In detail, VeroE6 cells were seeded in 24-well plates (9 × 10^4^ cells/well). The following day, serial dilutions of the viral stocks or tested supernatants were performed in serum-free DMEM media. After 1 h at 37 °C, overlay media was added to the inoculum to give a final concentration of 2% (v/v) FBS/DMEM media and 0.6% (v/v) methylcellulose (Merck Life science, Cat: M0512) to achieve a semisolid overlay. Plaque assays were incubated at 37 °C for 48 h. Samples were fixed using 5% Formaldehyde in PBS (Merck Life Science, Cat: 252549) and plaques were visualized using Crystal Violet solution (20% Ethanol, Merck Life science, Cat: C6158).

### Infection with SARS-CoV-2 Virus

Calu-3 cells (2.75 × 10^4^) were seeded in 96 well plates. HepG2 cells (1 × 10^5^) were seeded in 24 well plates. After 24 h, cells were infected with SARS-CoV-2 for 1 h at 37 °C at a MOI of 0.05, and mock controls were included in each experiment. After infection, cells were washed with PBS and fresh medium was added to each well. At 48 h, the medium was collected and the viral titer (expressed as PFU/ml) was calculated by plaque assay (PRA) in VeroE6 cells.

Infection experiments were performed with the SARS-CoV-2 isolate Milan IT (NCBI sequence MW000351.1), kindly provided by Prof. Cristiano Salata (Dept. of Molecular Medicine, University of Padova). The Omicron variant was provided by the Microbiology Unit of the Padua University Hospital and previously described (GenBank accession number: ON062195).

### Silencing Experiments

The genes of SB3, Protease-Activated Receptor 2 (PAR2) and TMPRSS2, as a positive control, together with unrelated genes used as negative controls, were silenced in Calu-3 cells by transient transfection. Cell reverse transfections were carried out using HiPerFect (Qiagen, 301704) (2.5 × 10^4^ cells/well in 96well format). The siRNAs were selected from the FlexiTube GeneSolution 4 siRNA sets (Qiagen, 102741) and transfected as a mix at 24 nM, following manufacturer’s instructions. Cells were harvested 48 h post-transfection, their total RNA was purified, retrotranscribed, and real-time PCR was performed.

For the evaluation of SARS-CoV-2 infectivity following silencing, the cell culture supernatant was removed and replaced with virus inoculum (MOI of 0.1), 24 h post siRNA transfection. Following 1 hr adsorption at 37 °C, the virus inoculum was removed and replaced with fresh 10% FBS DMEM/F-12 media. Cells were incubated at 37 °C for 48 h before supernatants were harvested. The viral titer (expressed as PFU/ml) was calculated by PRA in Vero E6 cells.

Primers used in the study are reported in Table S1.

### SerpinB3 Effect on the Protease TMPRSS2

In order to assess whether the antiprotease activity of SB3 was able to inhibit the protease TMPRSS2, whose relevance in favoring SARS-CoV-2 infection has been well established,^26^ experiments have been carried out using serial dilutions of recombinant SB3 protein, obtained in our laboratory, as previously described.^25^

### Kinetic Analysis

The activity of the recombinant TMPRSS2 was detected using the fluorescent Urokinase III substrate (Z-Gly-Gly-Arg-AMC,HCl)­(Sigma). TMPRSS2 (45 μL) was added to TBS (Tris-buffered saline, containing 50 nM Tris/HCl, 150 mM NaCl, pH 7.6) and 15 μL of 25 μM Z-Gly-Gly-Arg-AMC,HCl. The kinetic measurements were performed in a microtiter plate format using OptiPlate plates (Revvity MA, USA, ex PerkinElmer); the release of the fluorescent product was monitored recording the fluorescence emission at 455 nm using an excitation wavelength of 383 nm. All the kinetic measurements were run at 25 °C using a Victor X3 multilabel plate reader (PerkinElmer) controlled by the Workout 2.5 software (Revvity MA, USA, ex PerkinElmer). The raw fluorescence data were expressed as relative fluorescence units (rfu). Cleavage of the substrate by TMPRSS2 was also measured in the presence of a synthetic serine protease inhibitor Camostat mesylate (Merck) at 20 and 1 μM final concentration as the reference inhibitor.

### Kinetic Inactivation Studies

The kinetic analysis of the interaction between SB3 and TMPRSS2 was performed under first-order conditions using the progress curve method.^44^ To evaluate the antiprotease activity of SB3, inhibition experiments were performed using different concentrations of SB3 wild type (range = 0–10 μM). TMPRSS2 was mixed with increasing amounts of SB3, and the formation of the fluorescent product was monitored over time (300 s). The observed rate constant (K_obs_) values obtained at different concentrations of SB3 were plotted against the inhibitor concentration and the slope of this curve (k′), which represented the uncorrected second-order rate constant for the association between SB3 and TMPRSS2. The proper second-order rate constant was obtained correcting this value for the substrate concentration and the appropriate Km value, according to the equation Ka = k′ (1 + [S]_0_/Km).

### Analysis of Spike Binding to SB3

In order to explore the possible interaction of SB3 with the surface protein Spike, responsible for viral entry,^45^ different approaches were employed.

### Immunofluorescence

Since HepG2 cells were susceptible to SARS-Cov 2 infection only in the presence of SB3 with a conserved antiprotease activity, we assessed the possible binding of the viral protein Spike to the surface of HepG2 cells overexpressing the whole SB3 protein (HepG2/SB3) or to HepG2 cells transfected with the plasmid vector alone as control (HepG2/CTR). Recombinant trimeric Spike protein, obtained as recently reported,^46^ was used for immunofluorescence experiments. In detail, cells were fixed with 4% paraformaldehyde and blocked with 5% goat serum (Invitrogen Life Technologies, Waltham, MA, USA) in PBS containing 1% BSA, without permeabilization. Slides were incubated with monoclonal anti-Spike antibody obtained in rabbits (Sino Biological, Eschborn, Germany) and anti-SB3 antibody obtained in mice (Origene, Rockville, Maryland, USA) for 1 h at room temperature, followed by incubation with the Alexa-Goat 546 and 488 secondary antibodies (Invitrogen Life Technologies, Waltham, MA, USA), respectively. Cellular nuclei were counterstained with Dapi (Merck, Sigma-Aldrich, St. Louis, MO, USA). Slides were mounted with Fluoromount-G Mounting Medium (Invitrogen Life Technologies, Waltham, MA, USA) and observed under a fluorescence microscope (Axiovert 200M-Apotome.2, Carl Zeiss MicroImaging GmbH, Göttingen, Germany).

### Analysis of Spike/Protease Activated Receptor 2 Interaction

Since the RCL of SB3 was found to be essential for SARS-CoV2 infectivity, we addressed our attention to the Protease Activated Receptor 2 (PAR2). This membrane receptor was indeed recently found to be crucial for the induction of SB3 transcription, but only when the active loop of the protein is conserved. SB3 with its antiprotease activity determines an upregulation of this membrane receptor, in a positive loop manner.^19^ For this purpose, we have carried out immunofluorescence experiments to monitor the ability of Spike binding in HepG2 cells overexpressing SB3 that were silenced or not for PAR2. HepG2/SB3 cells were silenced by a transient transfection. Cell reverse transfections were carried out using Lipofectamine 3000 with 25 nM PAR2 siRNA (Santa Cruz Biotechnology, sc-36188, Dallas, Texas, USA) and negative control siRNA (Qiagen, Hilden, Germany). Forty-eight hours post-transfection, cells were treated with 7.5 μg/mL of trimeric Spike protein for 2 h. At this point immunofluorescence analysis was performed: cells were fixed with 4% paraformaldehyde and blocked with 5% goat serum (Invitrogen Life Technologies, Waltham, MA, USA) in PBS containing 1% BSA, without permeabilization. The expression of surface PAR2 and Spike was examined both in HepG2/SB3 cells silenced or not for PAR2 and in cells preincubated with Spike. As previously described, slides were incubated with monoclonal anti-Spike antibody obtained in rabbit (Sino Biological, Eschborn, Germany) and anti-SB3 antibody obtained in mouse (Origene, Rockville, Maryland, USA) for 1 h at room temperature, followed by incubation with Alexa-Goat 546 and 488 secondary antibodies, respectively (Invitrogen Life Technologies, Waltham, MA, USA). Cellular nuclei were counterstained with Dapi (Merck Sigma-Aldrich, St. Louis, MO, USA). Slides were mounted with Fluoromount-G Mounting Medium (Invitrogen Life Technologies, Waltham, MA, USA) and observed under a fluorescence microscope (Axiovert 200M-Apotome.2, Carl Zeiss MicroImaging GmbH, Göttingen, Germany).

To verify whether colocalization occurred, slides were observed under a confocal microscope (ZEISS LSM 900) and Pearson’s coefficient for signal overlapping was calculated using Zen3.9 software (Carl Zeiss MicroImaging GmbH, Göttingen, Germany).

### Functional Analysis

In order to assess whether PAR2 silencing affected SARS-CoV2 infectivity, we carried out experiments with two different viral strains (Wuhan and Omicron).

The effect of the exogenous addition of Spike on transcriptional expression of PAR2 was also explored in HepG2 cells overexpressing or not SB3 and in Calu-3, which are permissive to SARS-CoV2 infection. One x10^6^ cells were incubated with 7.5 ug/mL of trimeric Spike protein for 2 h, and RT-PCR amplification was carried out in cellular extracts, as described below.

In order to assess whether Spike protein could affect PAR2 activation in Calu-3 cells, phosphorylation levels of Erk1/2 (pErk1/2) were analyzed after Spike incubation, since MAPKs are widely known to belong to PAR2 signaling cascade.^47^ Western blot of Calu-3 cellular extracts, previously incubated for 2 h with Spike at 7.5 μg/mL concentration, and then subjected to activation of PAR2 using its agonistic peptide SLIGKV-NH2 (10 μM) for 10 min, were carried out as previously described.^29^

To further explore the involvement of the SB3 and PAR2 in Calu-3 cells, we have assessed the effect of SerpinB3 silencing or of the PAR2 inhibitor 1-PPA^29^ in relation to the expression of interferon-γ (IFN-γ), one of the major antiviral cytokines^48^ modulated by PAR2.^49^

In addition, we have also tested the cellular expression of Dipeptidyl-peptidase IV (DPPIV/CD26), a protease that was previously found to be induced by SB3 ^31^ and that more recently has been described to activate PAR2 signaling.^30^

### Western Blot Analysis

Cultured Calu-3 cells were first treated with trimeric Spike protein (7.4 μg/mL) for 2 h and subsequently treated with the SLIGKV-NH2 activating peptide (10 μM) for 10 min at 37 °C in 5% CO_2_ to allow PAR2 activation. Cellular extracts were loaded onto a linear gradient polyacrylamide gel (Invitrogen Bolt Bis-Tris Plus Mini Protein Gels, 4–12%) and electrophoresed in a Bolt MES SDS Running Buffer for 30 min at 200 V.

Protein bands were then transferred to a nitrocellulose membrane using the Power Blotter 1-Step Transfer Buffer (Thermo Fisher Scientific, Waltham, MA, USA), applying 25 V for 8 min at room temperature. Nitrocellulose membranes were blocked for 1 h in phosphate-buffered saline solution (PBS) containing 0.1% Tween-20 (TPBS) and 5% (w/v) bovine serum albumin (Merck Sigma-Aldrich, St. Louis, MO, USA).

Spike protein expression was detected using a rabbit monoclonal anti-Spike antibody (SinoBiological) diluted to 1:1000. Phosphorylation levels of Erk1/2 were assessed with an anti-pERK1/2 antibody (Cell Signaling, Danvers, MA, USA) diluted 1:1000, while total Erk1/2 was detected using the C-9 Erk1/2 antibody (Santa Cruz Biotechnology, Dallas, TX, USA) diluted 1:100. A mouse monoclonal anti-GAPDH antibody (Merck Sigma-Aldrich, St. Louis, MO, USA) diluted 1:1000 was used as the loading control. Anti-rabbit (Merck Sigma-Aldrich, St. Louis, MO, USA) and anti-mouse (KPL, SeraCare, Milford, MA, USA) HRP-conjugated antibodies were used as secondary antibodies. Bands were visualized and quantified using the chemiluminescent substrate LiteAblot Plus (Euroclone, Pero MI, Italy) and analyzed with an Alliance Q9 Atom (Uvitec, Cambridge, UK). All protein levels were normalized to those of the loading control.

### Real-Time Quantitative PCR

Transcriptional activity was assessed by quantitative real-time PCR (RT-PCR). In detail, total RNA was extracted from cultured cells using Trizol reagent (Invitrogen, Carlsbad, CA) according to the manufacturer’s instructions. After determination of total RNA purity and integrity, complementary DNA synthesis was carried out from 1 μg of RNA using LunaScript RT SuperMix (New England BioLabs). Quantitative RT-PCR reactions were performed according to the Luna Universal qPCR master Mix protocol (New England Biolabs, Ipswich, MA, USA), using the CFX96 Real-Time instrument (Bio-Rad Laboratories, Hercules, CA, USA). Relative gene expression was generated for each sample by calculating 2^–Δ*C*t^.^50^ The primer sequences used in the study are shown in Supplemental Table S1.

## Supplementary Material



## Notes

Figure S1. Nucleotide sequence position of SerpinB3 primers and SerpinB3 expression; Figure S2. SerpinB3 expression in Calu-3 and in HepG2 cells; Figure S3. Gene expression in Calu-3 in silenced cells; Figure S4. Kinetics of TMPRSS2 inhibition; Figure S5. Immunofluorescence colocalization analysis. Table S1. List and sequences of the primers used in the study. (PDF)
