# Supplementary material for: SerpinB3/Protease Activated Receptor‑2 Axis Is Essential for SARS CoV‑2 Infection
Source: ACS Infect Dis. 2025 May 23;11(9):2411–21. doi: 10.1021/acsinfecdis.5c00145 (PMC12442068; doi:10.1021/acsinfecdis.5c00145)
Supplement: Supplementary file 1 [file id5c00145_si_001.pdf]

**SUPPORTING INFORMATION**

# SERPINB3/PROTEASE ACTIVATED RECEPTOR-2 AXIS IS ESSENTIAL FOR SARS COV-2 INFECTION

Ilaria Frasson[1,*], Santina Quarta[2,*], Mariagrazia Ruvoletto[2], Alessandra Biasiolo[2], Monica Chinellato[2], Cristian Turato[3], Maristella Maggi[3], Laura Cendron[4], Sara N. Richter[1,**], Patrizia Pontisso[2,**]

[1]Dept of Molecular Medicine, University of Padua, via Gabelli 63, 35128 Padua, IT

[2]Dept of Medicine, University of Padua, via Giustiniani, 2, 35128 Padua, IT

[3]Dept of Molecular Medicine, University of Pavia, Viale Golgi 19, 27100 Pavia, IT

[4]Dept of Biology, University of Padua, via Bassi 58/B, 35121 Padua, IT

*These authors contributed equally to the study

** Shared senior authorship

**Corresponding Authors**:

Sara Richter (sara.richter@unipd.it); Patrizia Pontisso (patrizia@unipd.it)

# SUPPLEMENTAL FIGURES

```
ATGAATTCACTCAGTGAAGCCAACACCAAGTTCATGTTCGACCTGTTCCAACAGTTCAGAAAATCAAAAG
AGAACAACATCTTCTATTCCCCTATCAGCATCACATCAGCATTAGGGATGGTCCTCTTAGGAGCCAAAGA
CAACACTGCACAACAGATTAAGAAGGTTCTTCACTTTGATCAAGTCACAGAGAACACCACAGGAAAAGCT
GCAACATATCATGTTGATAGGTCAGGAAATGTTCATCACCAGTTTCAAAAGCTTCTGACTGAATTCAACA
AATCCACTGATGCATATGAGCTGAAGATCGCCAACAAGCTCTTCGGAGAAAAAACGTATCTATTTTTACA
GGAATATTTAGATGCCATCAAGAAATTTTACCAGACCAGTGTGGAATCTGTTGATTTTGCAAATGCTCCA
GAAGAAAGTCGAAAGAAGATTAACTCCTGGGTGGAAAGTCAAACGAATGAAAAAATTAAAAACCTAATTC
CTGAAGGTAATATTGGCAGCAATACCACATTGGTTCTTGTGAACGCAATCTATTTCAAAGGGCAGTGGGA
GAAGAAATTTAATAAAGAAGATACTAAAGAGGAAAAATTTTGGCCAAACAAGAATACATACAAGTCCATA
CAGATGATGAGGCAATACACATCTTTTCATTTTGCCTCGCTGGAGGATGTACAGGCCAAGGTCCTGGAAA
TACCATACAAAGGCAAAGATCTAAGCATGATTGTGTTGCTGCCAAATGAAATCGATGGTCTCCAGAAGCT
TGAAGAGAAACTCACTGCTGAGAAATTGATGGAATGGACAAGTTTGCAGAATATGAGAGAGACACGTGTC
GATTTACACTTACCTCGGTTCAAAGTGGAAGAGAGCTATGACCTCAAGGACACGTTGAGAACCATGGGAA
TGGTGGATATCTTCAATGGGGATGCAGACCTCTCAGGCATGACCGGGAGCCGCGGTCTCGTGCTATCTGG
AGTCCTACACAAGGCCTTTGTGGAGGTTACAGAGGAGGGAGCAGAAGCTGCAGCTGCCACCGCTGTAGTA
GGATTCGGATCATCACCTACTTCAACTAATGAAGAGTTCCATTGTAATCACCCTTTCCTATTCTTCATAA
GGCAAAATAAGACCAACAGCATCCTCTTCTATGGCAGATTCTCATCCCCGTAG
```

Reactive site loop (nt 1086-1148)

Δ-SerpinB3 deleted region (nt 1086-1109)

| Oligo | HepG2/CTR | HepG2/SB3 | HepG2/Δ–SB3 |
|---|---|---|---|
| SB3 | 2.87878E -08 | 1.11733E -05 | 0.000113748 |
| Δ–SB3 | 2.68458E -07 | 1.32864E -05 | 0 |

**Figure S1. Nucleotide sequence position of SerpinB3 primers and SerpinB3 expression.** Upper panel: Nucleotide sequence position of human SerpinB3 primers used in the study. The coding sequence (CDS) mRNA is derived from NCBI database (NM_006919, Nt 75-1247). In blu are indicated the primers used for the amplification of both SerpinB3-Wild-Type and Δ–SerpinB3 (Forward Nt 516-536; Reverse Nt 677-698), while in red are underlined the primers used for the amplification of only SerpinB3-Wild-Type (Forward Nt 1036-1054; Reverse Nt 1089-1107), since the forward primer overlaps the Δ-SB3 deleted region. Lower panel: the relative expression of SerpinB3 (SB3) mRNA in the different cell lines is reported and expressed as $2^{-\Delta CT}$.

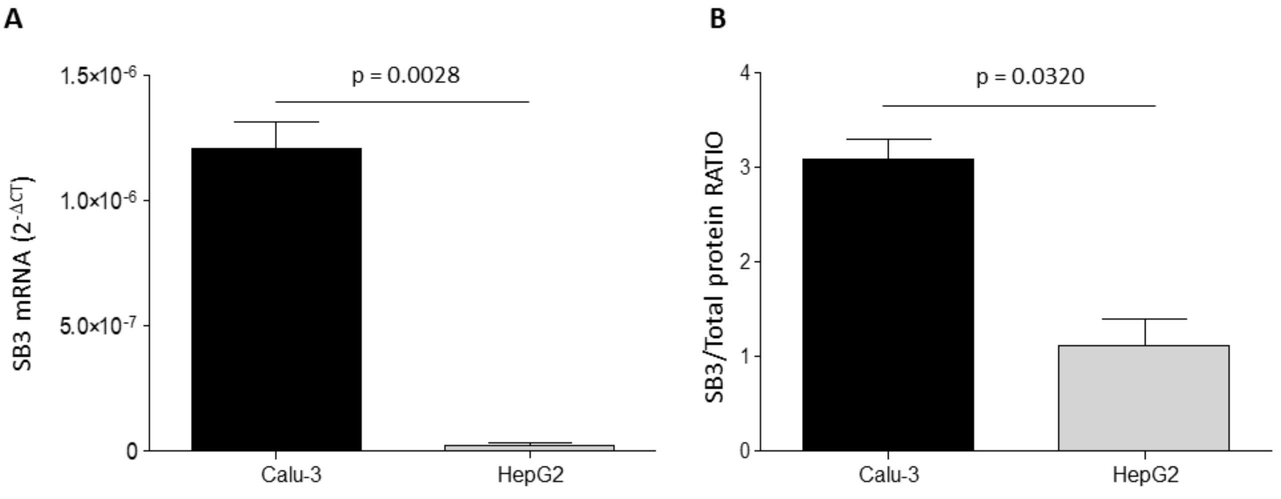


**Figure S2. SerpinB3 expression in Calu-3 and in HepG2 cells.** A) Relative SerpinB3 (SB3) mRNA expression levels measured by real-time PCR are expressed as $2^{-\Delta Ct}$. B) Protein expression was measured by ELISA and reported as the ratio between SerpinB3 protein and the total protein level. Bars represent the mean results ± SD of three different experiments (Mann-Whitney test).

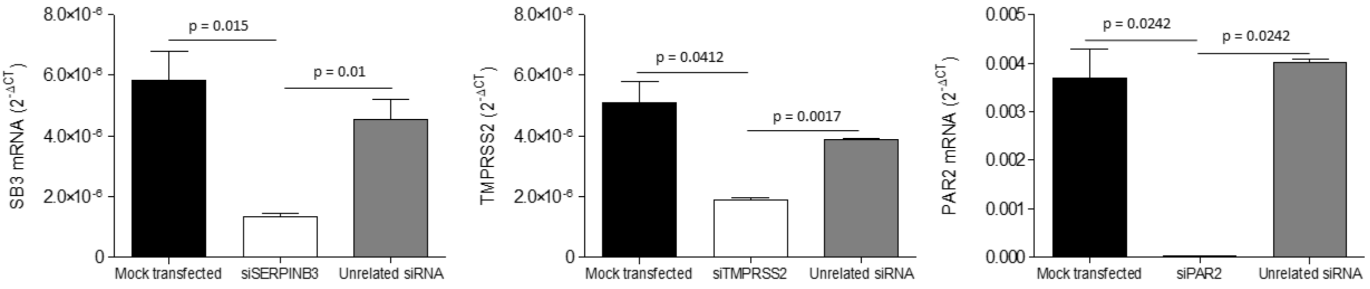


**Figure S3**. **Gene expression in Calu-3 in silenced cells**. Relative mRNA expression levels of SerpinB3, TMPRSS2 and PAR2 in silenced cells were assessed by real-time PCR and expressed as $2^{-\Delta Ct}$. Mock transfected cells and cells transfected with unrelated siRNA were used as negative controls. Bars represent the mean results ± SD of three different experiments (Mann-Whitney test).

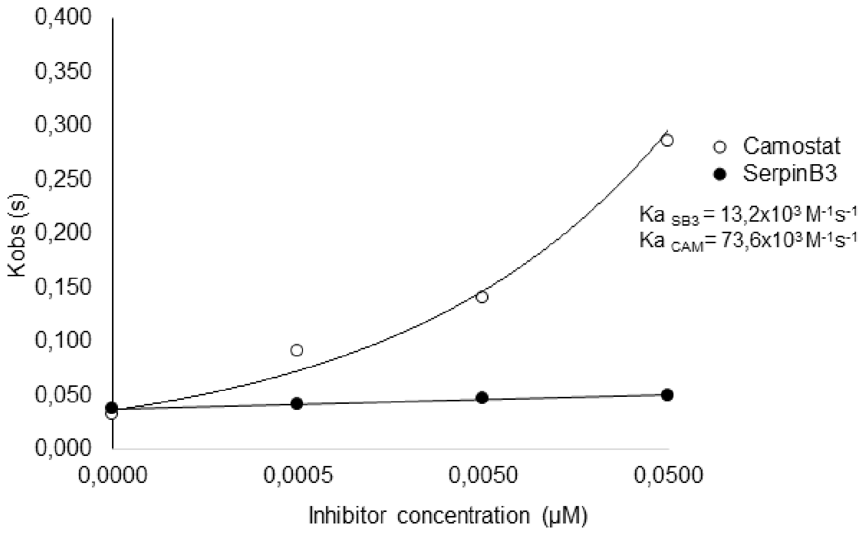


**Figure S4. Kinetics of TMPRSS2 inhibition.** The effect of different concentrations of SerpinB3 and of the reference TMPRSS2 inhibitor Camostat was carried out analysing the activity of TMPRSS2, measured as the fluorescence emission at 455 nm of the fluorescent Urokinase III substrate (Z-Gly-Gly-Arg-AMC,HCl).
$K_{obs,}$ observed rate constant; Ka $_{SB3}$, SB3 constant; Ka $_{CAM}$ , Camostat constant.

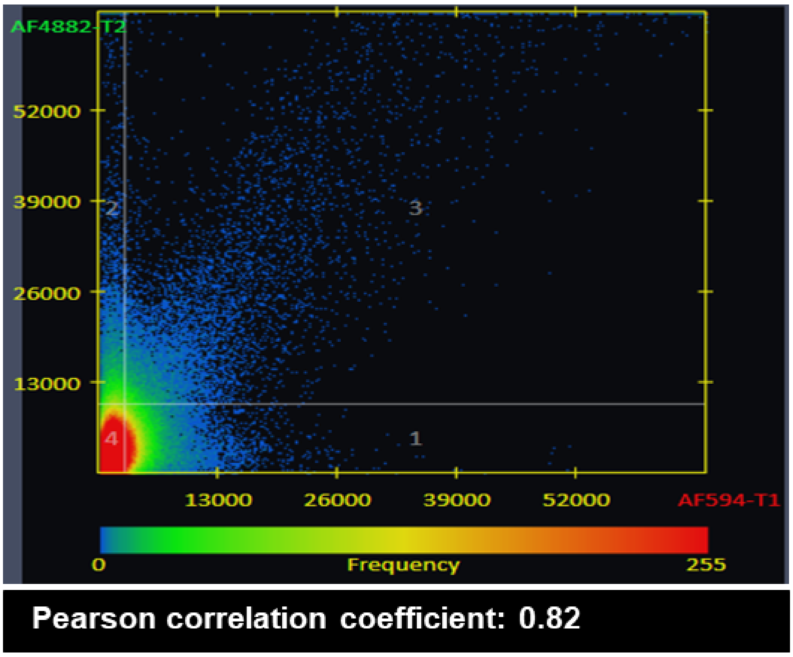


**Figure S5. Immunofluorescence co-localization analysis.** Spike and SerpinB3 were immune stained in unpermeabilized HepG2 cells overexpressing SerpinB3 and observed under confocal microscope. Pearson's correlation coefficient for signal overlapping was obtained using Zen.3.9 software.

## SUPPLEMENTAL TABLE

**Table S1**. List and sequences of the primers used in the study.

| Gene | Forward | Reverse |
|---|---|---|
| **45S** | 5’-CGGCTACCACATCCAAGGAA-3’ | 5’-GCTGGAATTACCGCGGCT-3’ |
| **SerpinB3** | 5’-AACTCCTGGGTGGAAAGTCAA-3’ | 5’-ACCAATGTGGTATTGCTGCCAA-3’ |
| **SerpinB3 Δ7** | 5’-GCGGTCTCGTGCTATCTGG-3’ | 5’-CTGCAGCTTCTGCTCCCTC-3’ |
| **PAR-2** | 5’ GCTAGCAGCCTCTCTCTCCT 3’ | 5’-GTGGGATGTGCCATCAACCT-3’ |
| **DPP-IV** | 5’-GGCACCTGGGAAGTCATCGGG-3’ | 5’-CCGGATTCAGCTCACAACTGAGGC-3’ |
| **γ-INF** | 5’- GTGGAGACCATCAAGGAAGACA-3’ | 5’-CTGGCGACAGTTCAGCCATC-3’ |
| **IL-6** | 5’-AGTGAGGAACAAGCCAGAGC-3’ | 5’-GTCAGGGGTGGTTATTGCAT-3’ |
| **CCL-2** | 5’-CCCCAGTCACCTGCTGTTAT-3’ | 5’-AGATCTCCTTGGCCACAATG-3’ |
| **TNF-α** | 5’-AACCTCCTCTCTGCCATCAA-3’ | 5’-GGAAGACCCCTCCCAGATAG-3’ |
| **IL-1β** | 5’-TGAAAGCTCTCCACCTCCAG-3’ | 5’-CACGCAGGACAGGTACAGAT-3’ |
